# Delayed involution of lactation presenting as a non-resolving breast mass: a case report

**DOI:** 10.1186/1752-1947-2-327

**Published:** 2008-10-13

**Authors:** Yashwant Kumar, Alka Chahal, Monika Garg, Alka Bhatia, NC Mahajan, Anil Ganju

**Affiliations:** 1 Department of Pathology, Maharshi Markandeshwar Institute of Medical Sciences and Research, Mullana, Ambala Haryana, India; 2 Department of General Surgery, Maharshi Markandeshwar Institute of Medical Sciences and Research, Mullana, Ambala Haryana, India

## Abstract

**Introduction:**

Involution of lactation is a physiological process. Rarely, it may be delayed and troublesome for the lactating woman. Though lactation-induced changes in breast are well known, morphological features of delayed involution are not clear.

**Case presentation:**

We report a case of a 22-year-old lactating mother who presented with a painful, non-resolving breast mass 5 months after delivery. Clinically, it simulated an inflammatory carcinoma. Histopathology, however, revealed involuting lactational changes.

**Conclusion:**

To the best of our knowledge, lactational involution with such a presentation has not been described in the English literature. The case needs to be reported so that this entity can be considered among the differential diagnoses of breast masses in a lactating patient.

## Introduction

The sequence of events involved in lactation and its involution have been well studied in animals but the data in humans are scanty [1]. The involution of lactation is a process by which a fully functional, lactating mammary gland regresses to a quiescent, resting stage. This process of involution is a multistage process [2], usually begins after 3 months of lactation and is symptomless [3]. It begins with the onset of cessation of milk secretion, during which the acinar cells lose their differentiated phenotype. This is followed by collapse of the acini with apoptosis of acinar cells and clearance of residual milk by the phagocytes. Finally, there is re-growth of the stromal adipose tissue resulting in morphology similar to the postpubertal nonlactating gland. Rarely, the process of involution may be delayed and failure to remove unnecessary lactational acini may result in symptomatic inflammatory tissue damage. The morphological features of delayed involution are not clearly defined. To the best of our knowledge, this is the first report describing the morphology of delayed lactational involution which presented as a non-resolving breast mass.

## Case presentation

A 22-year-old woman who was 5 months postpartum presented with a left-sided breast lump of 3 months duration. The lump was associated with pain and tenderness. For the last 15 days, she had also developed retraction of nipple along with thick nipple discharge. This was her first pregnancy and she could not feed her child on this side. There was no history of fever or trauma to the breast. On examination, her left breast was grossly enlarged with red and inflamed overlying skin. The nipple-areola complex was markedly enlarged with hyperpigmentation of areola but there was no excoriation. Thick discharge was seen on the nipple. On palpation, a large, multinodular, tender lump of about 6.0 × 5.0 × 2.4 cm was felt in the upper and outer quadrant. The lump was firm to hard in consistency. It was free from the chest wall and the overlying skin. The opposite breast was normal. A clinical possibility of inflammatory carcinoma was suspected and the patient was sent for fine needle aspiration cytology (FNAC). This confirmed a benign inflammatory lesion. A course of 2 weeks of antibiotics was given. However, the lump persisted following which it was excised and sent for histopathology. The postoperative period was uneventful. At present, both mother and baby are healthy and the baby is feeding from the right breast.

**Figure 1:**
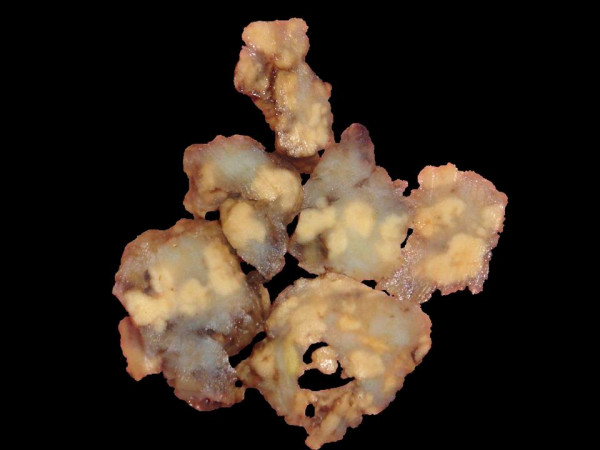
**Gross photograph of the specimen removed in piecemeal.** The cut surface shows multiple well circumscribed nodules some of which are confluent. These nodules are solid and yellowish in colour and are separated by thick translucent areas.

Grossly, the specimen was received in the form of multiple irregular tissue pieces measuring 0.8 × 08 × 1.0 cm to 4.0 × 2.5 × 1.5 cm in size. The outer surface was lobulated and the cut surface showed multiple yellowish, soft nodules ranging from 0.3 to 1.0 cm in diameter. The margins of each nodule were circumscribed and well demarcated from the surrounding stroma. The adjacent areas were whitish and translucent (Figure 1).

**Figure 2:**
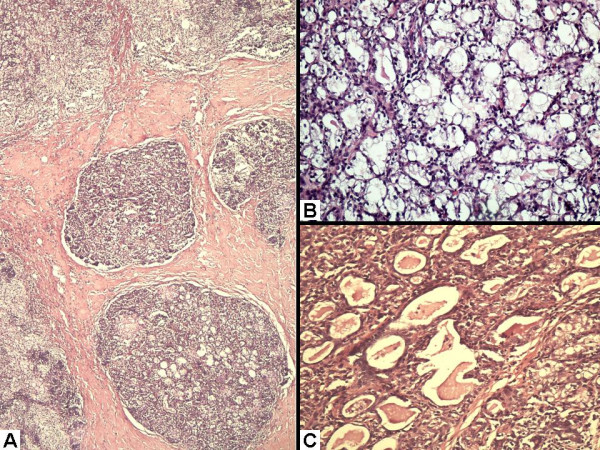
**Haematoxylin and eosin stained sections showing lactational changes: **(A) Low power view to show two types of lobule. Note the extensive hyalinization of surrounding stroma. (B) Some of the lobules showed acini lined with epithelial cells with abundant vacuolated cytoplasm. (C) Dilated ducts within the lobules containing eosinophilic secretions.

**Figure 3:**
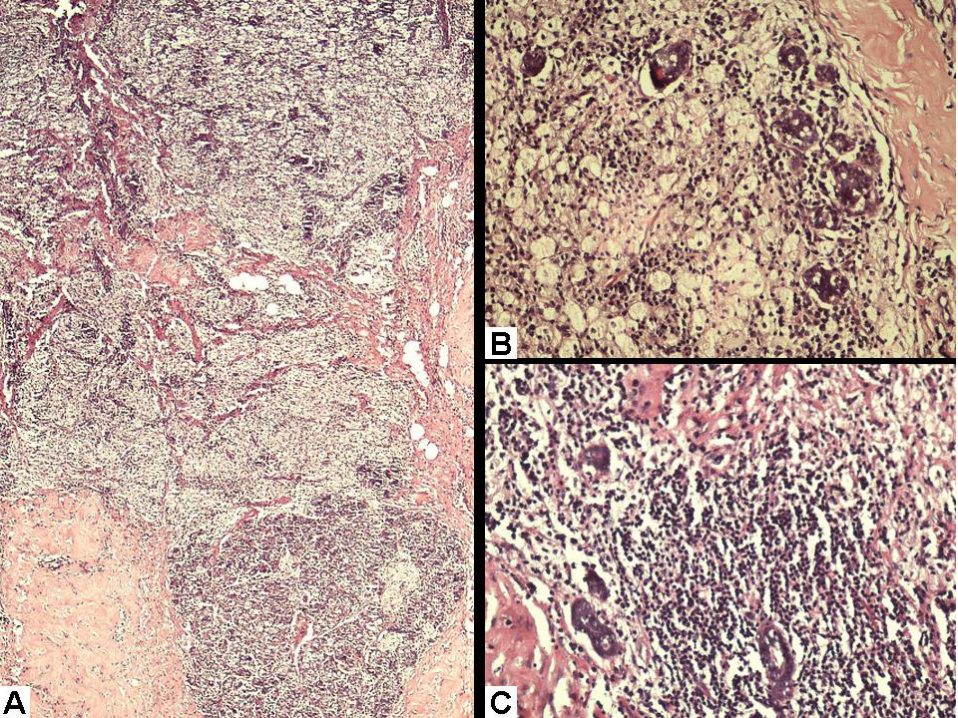
**Haematoxylin and eosin stained sections showing:** (A) Sheets of macrophages infiltrating the lobules and extending into the adjacent stroma. (B) High power view depicting macrophages destroying one of the breast lobules. Only a few acini are preserved. (C) Lymphocytic aggregate within the lobule.

Microscopic examination revealed two types of lobules separated by an extensively hyalinized stroma (Figure 2A). Some of these lobules showed classical lactational changes comprising many hyperplastic and dilated acini filled up with pink, eosinophilic secretions. These acini were lined with round to oval cells with fine chromatin and conspicuous nucleoli. Many of these cells had granular eosinophilic cytoplasm. Others had abundant, clear vacuolated cytoplasm with the nucleus pushed to the periphery (Figure 2B). The ducts were also dilated and filled up with milk (Figure 2C). The second type of lobules depicted ongoing involutional changes in the form of sheets of macrophages replacing and destroying the acini (Figure 3A and 3B). In places, the phagocytic cells showed prominent admixture with lymphocytes some of which were also found infiltrating into the acinar epithelium. A few lymphoid aggregates were also noted around the individual ducts and acini (Figure 3C). Based on all these findings, a diagnosis of lactating breast with delayed involution was offered.

## Discussion

During pregnancy and lactation, the breast is subjected to a variety of morphological changes. These range from hyperplastic lobules filled with milk containing ducts and acini to destruction of these acini when the job is done. The latter process is known as involution and is accomplished by means of increased apoptosis of epithelial cells and phagocytosis of the residual milk and cell debris. This is followed by stromal regenerative changes. All of these changes prepare the breast for the next pregnancy [4]. Any delay in involution of lactation may lead to troublesome symptoms.

Delayed involution is characterized by a peculiar morphology. The breast shows a mixed picture comprising both hyperplastic and involuting lobules along with infiltration by inflammatory cells. The polymorphs and macrophages are involved in phagocytosis and clearance of unwanted acini. The lymphocytes are probably involved in the transfer of immunological agents from mother to offspring via the milk during lactation but their role in involution is not known. Focal calcification may be seen [5], however this was absent in our patient.

The condition may clinically or morphologically mimic many other diseases which occur during lactation such as mastitis, galactocele, lactating adenoma and rarely inflammatory carcinoma. Lactation mastitis is characterized by a heavy polymorphonuclear infiltrate due to infection acquired during feeding of the baby. A galactocele is a cystic dilatation of a breast duct as a result of obstruction by inspissated secretions. Another lesion which may simulate the delayed involution is a nodular lactating adenoma. This neoplastic lesion can be easily differentiated by its gross circumscription, lack of inflammation and scanty stroma [6]. Inflammatory carcinoma is a close differential clinically, but does not pose any difficulty in diagnosis on microscopy.

The pathophysiology of lactational involution has been extensively studied in animals. The process is presumably controlled by lowered milk demand, decreased milk removal and slow changes in the circulating hormones and insulin-like growth factors (GF-I, IGFBP-3) [7]. Several molecular factors including AKT1/Protein kinase Bα, P53, and stat3 have also been implicated in mammary involution [8,9]. It is possible that abnormal expression of one or more genes regulating these chemical substances may be responsible for delayed involution. Further studies on cases like ours may provide a definite causal link between these factors and delayed involution.

Studies in the past have demonstrated the protective role of breast feeding in mammary carcinoma [10]. One mechanism postulated for this effect is the apoptosis of abnormal cells at early stages of involution [11]. Some of the more recent studies have shown the importance of components of the fatty stroma of involuting breast in metastasis [12]. In this patient, the delay in involution was accompanied by a low apoptotic activity and lack of fatty stroma. The significance of these findings, if any, in pathogenesis of breast cancer should be studied further in larger series of patients. Therefore, more cases like ours need to be reported.

The treatment protocol in symptomatic delayed involution of lactation is not well characterized. However, in our patient, surgical excision provided symptomatic relief.

## Conclusion

This report documents the rare occurrence of symptomatic delayed involution of lactation presenting as a non-resolving breast lump. It is important for pathologists and clinicians to be aware of such an entity to distinguish it from clinical and morphological mimics, so that a definitive therapy can be planned.

## Competing interests

The authors declare that they have no competing interests.

## Consent

Written informed consent was obtained from the patient for publication of this case report and any accompanying images. A copy of the written consent is available for review by the Editor-in-Chief of this journal.

## Authors' contributions

YK was primarily responsible for drafting of the manuscript, literature search and diagnosis of the case. AC and MG both have made critical revision for important intellectual content. AB diagnosed the case, undertook proofreading, corrections in the manuscript and final submission. NCM gave final approval of the version to be published, AG provided the relevant clinical data. All authors have read and approved the final manuscript.
